# Multiple m^6^A RNA methylation modulators promote the malignant progression of hepatocellular carcinoma and affect its clinical prognosis

**DOI:** 10.1186/s12885-020-6638-5

**Published:** 2020-02-28

**Authors:** Nanfang Qu, Sanyu Qin, Xuemei Zhang, Xiaotong Bo, Zhengchun Liu, Chao Tan, Guiqiong Wen, Haixing Jiang

**Affiliations:** 1 Department of Gastroenterology, The First Affiliated Hospital of Guangxi Medical University, Nanning, 530021 China; 2 Department of Gastroenterology, Affiliated Hospital of Guilin Medical University, Guilin, 541000 China; 3 Department of Pathology, Affiliated Hospital of Guilin Medical University, Guilin, 541000 China; 4 Department of Radiotherapy, Affiliated Hospital of Guilin Medical University, Guilin, 541000 China; 5 School of Public Health, Guilin Medical University, Guilin, 541000 China; 6 Department of B Ultrasound, Affiliated Hospital of Guilin Medical University, Guilin, 541000 China

**Keywords:** m6A, RNA methylation, Liver hepatocellular carcinoma, Prognosis

## Abstract

**Background:**

Hepatocellular carcinoma (HCC) is the second most common cause of cancer-related death in the world. N^6^-methyladenosine (m^6^A) RNA methylation is dynamically regulated by m^6^A RNA methylation modulators (“writer,” “eraser,” and “reader” proteins), which are associated with cancer occurrence and development. The purpose of this study was to explore the relationships between m^6^A RNA methylation modulators and HCC.

**Methods:**

First, using data from The Cancer Genome Atlas (TCGA) and International Cancer Genome Consortium (ICGC) databases, we compared the expression levels of 13 major m6A RNA methylation modulators between HCC and normal tissues. Second, we applied consensus clustering to the expression data on the m^6^A RNA methylation modulators to divide the HCC tissues into two subgroups (clusters 1 and 2), and we compared the clusters in terms of overall survival (OS), World Health Organization (WHO) stage, and pathological grade. Third, using least absolute shrinkage and selection operator (LASSO) regression, we constructed a risk signature involving the m^6^A RNA methylation modulators that affected OS in TCGA and ICGC analyses.

**Results:**

We found that the expression levels of 12 major m6A RNA methylation modulators were significantly different between HCC and normal tissues. After dividing the HCC tissues into clusters 1 and 2, we found that cluster 2 had poorer OS, higher WHO stage, and higher pathological grade. Four m^6^A RNA methylation modulators (YTHDF1, YTHDF2, METTL3, and KIAA1429) affecting OS in the TCGA and ICGC analyses were selected to construct a risk signature, which was significantly associated with WHO stage and was also an independent prognostic marker of OS.

**Conclusions:**

In summary, m^6^A RNA methylation modulators are key participants in the malignant progression of HCC and have potential value in prognostication and treatment decisions.

## Background

According to the Cancer Statistics of China in 2015, hepatocellular carcinoma (HCC) is the second and fifth most common cause of cancer-related death in men and women, respectively [1].Worldwide, HCC is the most common type of primary liver cancer, ranking second among malignant tumors, with more than 700,000 deaths each year [2]. Chronic liver inflammation caused by hepatitis B virus (HBV), hepatitis C virus (HCV), or aflatoxin B1 is the main cause of HCC [3]. A series of treatments have been developed for patients with HCC, including surgery, chemotherapy, and radiotherapy. However, the mortality rate is still very high [4–6]. Understanding the molecular mechanisms underlying the development of HCC is essential to improve diagnostic tools and treatments.

RNA methylation in mammalian cells is dynamic and reversible and has been suggested as another kind of epigenetic regulation analogous to DNA methylation or histone modification [1, 7]. N^6^-methyladenosine (m^6^A) is considered the most frequent, abundant, and conserved internal modification of eukaryotic messenger RNA (mRNA) [8], microRNAs (miRNAs) [9], and long non-coding RNA [10]. The m^6^A modification occurs at a consistent motif, RRm^6^ACH([G/A/U][G > A]m^6^AC[U > A > C]) [11]. In addition, experiments using m^6^A-specific antibodies and high-throughput sequencing (HTSeq) revealed that m^6^A modifications mainly appear around termination codons, in 3′-untranslated regions, and in long internal exons [12]. These modifications play key roles in 3′-end processing, pre-RNA splicing, translation regulation, nuclear output, miRNA processing, and RNA attenuation [13].

The m^6^A modification can be reversed in a process coordinated by methyltransferases (m6A “writers”), demethylases (m^6^A “erasers”), and m^6^A “reader” proteins [14]. The m^6^A “writer” complex is composed of METTL3, WTAP, RBM15, METTL14, VIRMA (KIAA1429), and ZC3H13 [13, 15]. So far, only FTO and ALKBH5 have been identified as m^6^A “erasers” [16]. As for m^6^A “readers,” including YTHDF1–2, YTHDC1–2, and HNRNPC, they can recognize m^6^A modifications and consequently direct RNA alternative splicing, localization, translation, and stability, among other processes [17, 18]. Thus, m^6^A modifications direct mRNAs to different destinies through differential processing, translation, and decay during the stress response, embryonic development, and cell differentiation [19]. Increasing evidence has shown that genetic changes (e.g., copy number variation [CNV] and single-nucleotide polymorphism [SNP] mutations) and expression disorders related to m^6^A RNA methylation modulators are closely related to the malignant progression of various cancers [20–24]. Many studies have pointed out that abnormal m^6^A modification is associated with HCC progression [20, 25–29]. However, the role of m^6^A RNA methylation modulators in the malignant progression of HCC and their impact on prognosis has not been fully analyzed.

In this study, the expression in HCC tissues of 13 widely reported m^6^A RNA methylation modulators was systematically analyzed using RNA sequencing data from The Cancer Genome Atlas (TCGA) and the International Cancer Genome Consortium (ICGC). Additionally, CNV and SNP mutations of these 13 modulators in the TCGA database were explored. According to the expression of m^6^A RNA modulators, the HCC tissues were divided into clusters 1 and 2, and there were differences in the clinicopathological factors between the two groups. Furthermore, four m^6^A RNA methylation modulators were selected to construct a least absolute shrinkage and selection operator (LASSO) risk regression model (a four-gene risk signature) to predict HCC prognosis in terms of survival.

## Methods

### Datasets

RNA sequencing transcriptome data and corresponding clinicopathological data were acquired for 374 HCC and 50 normal tissues from TCGA (http://cancer.gov/) and 243 HCC and 202 normal tissues from ICGC (https://dcc.icgc.org/releases/current/Projects/LIRI-JP). We used Liver Cancer, RIKEN, Japan [LIRI-JP] data, because the Chinese datasets are incomplete. For the TCGA transcriptome analysis, we downloaded HTSeq-fragments per kilobase of transcript per million mapped reads (FPKM) data. Clinicopathological data from the TCGA and ICGC datasets are presented in Table S1. We deleted the TCGA data on all samples with incomplete data regarding age, sex, World Health Organization (WHO) stage, and pathological grade in the analyses exploring the relationships between clinicopathological factors and HCC subtype (cluster 1 or 2) and between clinicopathological factors and the risk score; this led to *n* = 350 HCC samples in these TCGA analyses. In the survival analysis, we deleted the data on samples with “futime” variable (survival or censoring time) = 0; this led to *n* = 371 HCC samples in the TCGA analysis.

Regarding the CNV and SNP analyses, we used TCGA data. Using the official TCGA website, we entered the Genomic Data Commons (GDC) Data Portal and selected “Liver” as the Primary Site and “TCGA-LIHC” as the Project. By downloading the Copy Number Variation in the Masked Copy Number Segment data type from the TCGA database, CNV data were obtained, comprising data on 379 HCC and 389 normal tissues. By downloading the Simple Nucleotide Variation of Masked Somatic Mutation data type from the TCGA database, SNP data were obtained, and then we analyzed these data that included 364 HCC tissues (TCGA.LIHC.varscan.40fe9c1b-19d0-45cf-898a-f7b0cbad783e.DR-10.0.somatic.maf file).

### Bioinformatics analysis

We used ActivePerl 5.24.3 Build 2404 (https://www.perl.org), R v3.6.0 (https://www.r-project.org/), and SPSS v23.0 (IBM Corp., Armonk, NY, USA) to conduct data conversion, statistical analysis, and calculations. All our R and Perl packages were obtained from the Shengxin Self-learning Network (https://www.biowolf.cn/).

To compare the expression of 13 genes encoding m^6^A RNA methylation modulators between HCC and normal tissues, we used the R package “pheatmap.”

To investigate the function of m^6^A RNA methylation modulators in HCC, we clustered the HCC tissues in the TCGA dataset into two groups using the “ConsensusClusterPlus” and “limma” packages. We used the principal component analysis “PCA” package to explore the feasibility of tumor typing based on m^6^A-related gene expression.

To explore the prognostic value of m^6^A RNA methylation modulators, univariate Cox regression analyses were performed using the expression levels of the 13 genes in the TCGA dataset. Thus, we identified nine genes that were significantly related to survival (*P* < 0.05), and further we used a LASSO regression to narrow the range of target genes because the predictor variable was much larger than the sample content in the gene expression data [30]. Finally, five genes were obtained.We took the same approach using ICGC data and obtained seven genes. We then selected the four genes that were common to both analyses for further analysis. We conducted another LASSO analysis of the four genes and selected the best penalty parameter (λ). We obtained the same results using the two databases. Finally, the four genes were used to construct a risk signature. The risk score based on the signature was calculated using the following formula [31]:

$$ \mathrm{Risk}\ \mathrm{score}=\sum {\mathrm{n}}_{\mathrm{i}}=\sum \left(\mathrm{Coefi}\times {\mathrm{x}}_{\mathrm{i}}\right) $$

where Coef_i_ is the coefficient and x_i_ is the z-score-transformed relative expression value of each selected gene. We used this formula to calculate a risk score for each patient in both the training (TCGA) and validation (ICGC) datasets.

To explore the prognostic value of the four-gene risk signature, first, we combined gene expression data with survival status and time data. HCC patients in the TCGA (*n* = 365) and ICGC (*n* = 232) datasets were divided into low- and high-risk groups based on the median risk score, and we compared the overall survival (OS) rate between the two groups using the Kaplan–Meier method.We also constructed nomograms based on the risk score and clinicopathological factors to predict 1-, 3-, and 5-year survival rates, using the R packages “Hmisc,” “lattice,” “Formula,” “ggplot2,” “foreign,” and “rms,” Finally, we verified the results sing the ICGC data.

### Statistical analysis

To identify differential expression between HCC and normal tissues, the homogeneity of variance assumption was assessed and found not to be satisfied, so Wilcoxon rank-sum tests were used rather than t tests. The chi-square test was used to compare CNV variation rates between the normal and tumor groups. For comparison of CNV among multiple groups (i.e., one copy lost, normal, one copy gained, and two or more copies gained), the homogeneity of variance assumption was assessed and found not to be satisfied, so Kruskal–Wallis tests were used rather than one-way analysis of variance.

After clustering the patients into two clusters based on consensus expression of the m^6^A RNA methylation modulators, we used chi-square tests to compare the distribution of age, sex, WHO stage, and pathological grade between clusters 1 and 2 [31].

The Kaplan–Meier method and bilateral log-rank test were used to compare OS between HCC subtypes cluster 1 and 2 and between high- and low-risk groups (based on the median risk score calculated using the four-gene risk signature). The prediction efficiency of the risk score for 3-year survival was assessed using a receiver operating characteristic (ROC) curve analysis.

Univariate Cox regression analyses were used to assess the associations between the expression levels of the 13 genes and OS. Additionally, uni- and multivariate Cox regression analyses were used to determine the prognostic values of the risk score and various clinicopathological factors.

## Results

### Most m^6^A RNA methylation modulators are upregulated in HCC

**Fig. 1:**
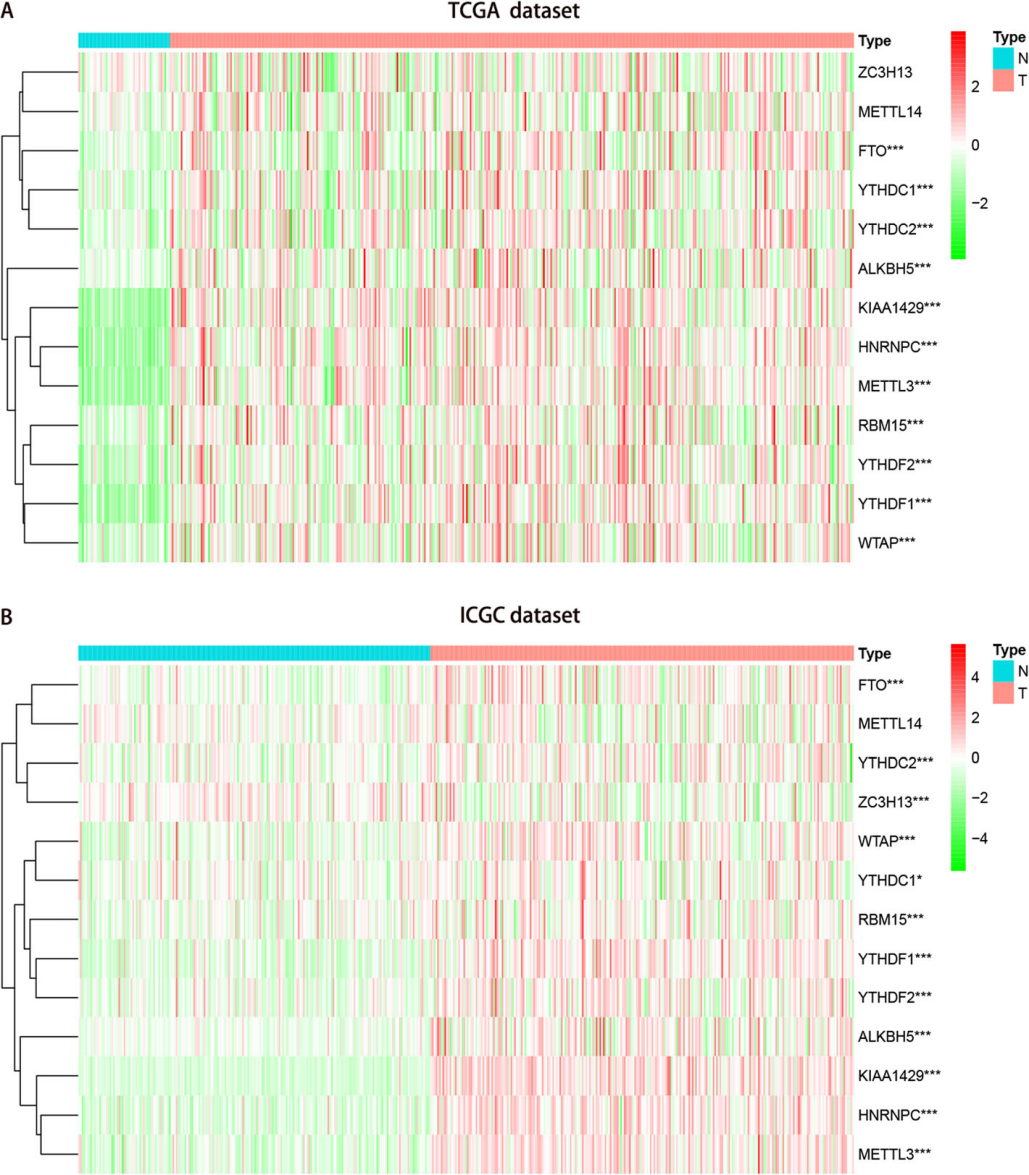
Expression of m6A RNA methylation modulators in hepatocellular carcinoma. **a** Data from TCGA dataset; **b** Data from ICGC dataset; Wilcox test was used to determine the differential gene expression between tumor group and normal group. * *P* < 0.05 and *** *P* < 0.001

TCGA transcriptome data showed that all 13 genes except for ZC3H13 and METTL14 were differentially highly expressed in HCC tissues compared with normal tissues (Fig. 1A, Table S1). Further verification using ICGC data showed that all 13 genes except for METTL14 were differentially highly expressed in HCC tissues compared with normal tissues (Fig. 1B, Table S1).

### CNV and SNP analyses regarding m^6^A RNA methylation modulators in HCC

To understand whether the differential expression of the m^6^A RNA methylation modulators is caused by genetic changes related to the corresponding genes, we analyzed the CNV and SNP data from the TCGA database. First, we found that the CNV of the 13 genes in HCC tissues was significantly different from that in normal tissues. More specifically, three genes (YTHDF1, YTHDC2, and KIAA1429) mainly exhibited increased copy numbers in HCC tissues, while the remaining 10 genes mainly exhibited decreased copy numbers (Figure S1A, Table S1). Second, we explored the relationships between mRNA expression and copy number and found that all 12 genes upregulated in the TCGA analysis except for HNRNPC exhibited significantly increased copy numbers (Figure S2).

Furthermore, we observed that SNP mutations in the 13 genes were very low (all ≤1.9%) in HCC tissues (Figure S1B). However, as mentioned earlier, the expression of most m^6^A RNA methylation modulators was increased in HCC tissues compared to normal tissues, indicating that these changes in gene expression are not entirely caused by CNV or SNP mutations in the corresponding genes.

### Consensus clustering of m6A RNA methylation modulators identified two clusters of HCC tissues which were associated with clinicopathological factors

**Fig. 2:**
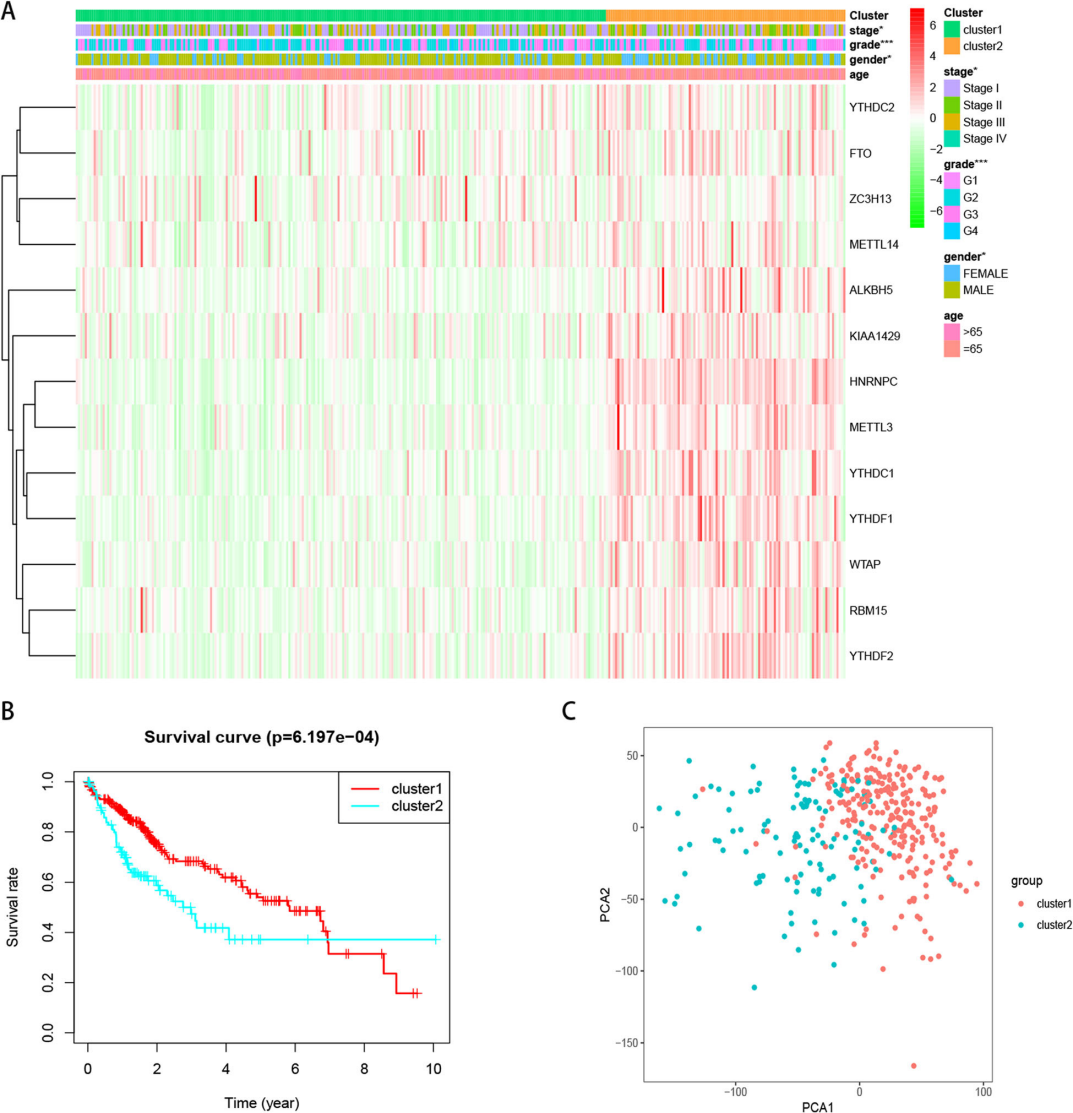
Differential clinicopathological factors and overall survival of hepatocellular carcinoma in the cluster 1/2 subgroups. **a** Heatmap and clinicopathological factors of the two clusters, Chi-square test was used for correlation between clinical and cluster, * *P* < 0.05 and *** *P* < 0.001. **b** Kaplan–Meier overall survival (OS) curves for 374 TCGA hepatocellular carcinoma patients. The sample size of cluster 1 and cluster 2 is 257 and 117 respectively. **c** Principal component analysis of the m6A-related gene expression in the TCGA dataset, hepatocellular carcinoma in the cluster1 subgroup are marked with red

Based on the similarity of expression levels of the m6A RNA methylation modulators, we observed the clustering stability of the TCGA dataset from k = 2 to 9 (Figures S3A–E). It can be seen that k = 2 seems to be an appropriate choice. Subsequently, we analyzed the differential gene expression of the two subgroups, designated as clusters 1 and 2, and found that the expression of m6A RNA methylation modulators was higher in cluster 2 than in cluster 1 (Figure S3F). We then compared the clinicopathological factors between the two clusters. Cluster 1 was significantly associated with male sex (*P* < 0.05), lower pathological grade (*P* < 0.001), and lower WHO stage (*P* < 0.05), while cluster 2 was significantly associated with female sex, higher pathological grade, and higher WHO stage (Fig. 2A). We further found that cluster 2 had significantly shorter OS than cluster 1 (Fig. 2B). Next, we used PCA to explore the feasibility of tumor typing based on m6A-related gene expression. The results showed that there were significant differences between the two tumor subtypes and tumor typing was feasible (Fig. 2C).

### Prognostic value of m^6^A RNA methylation modulators and risk signature

**Fig. 3:**
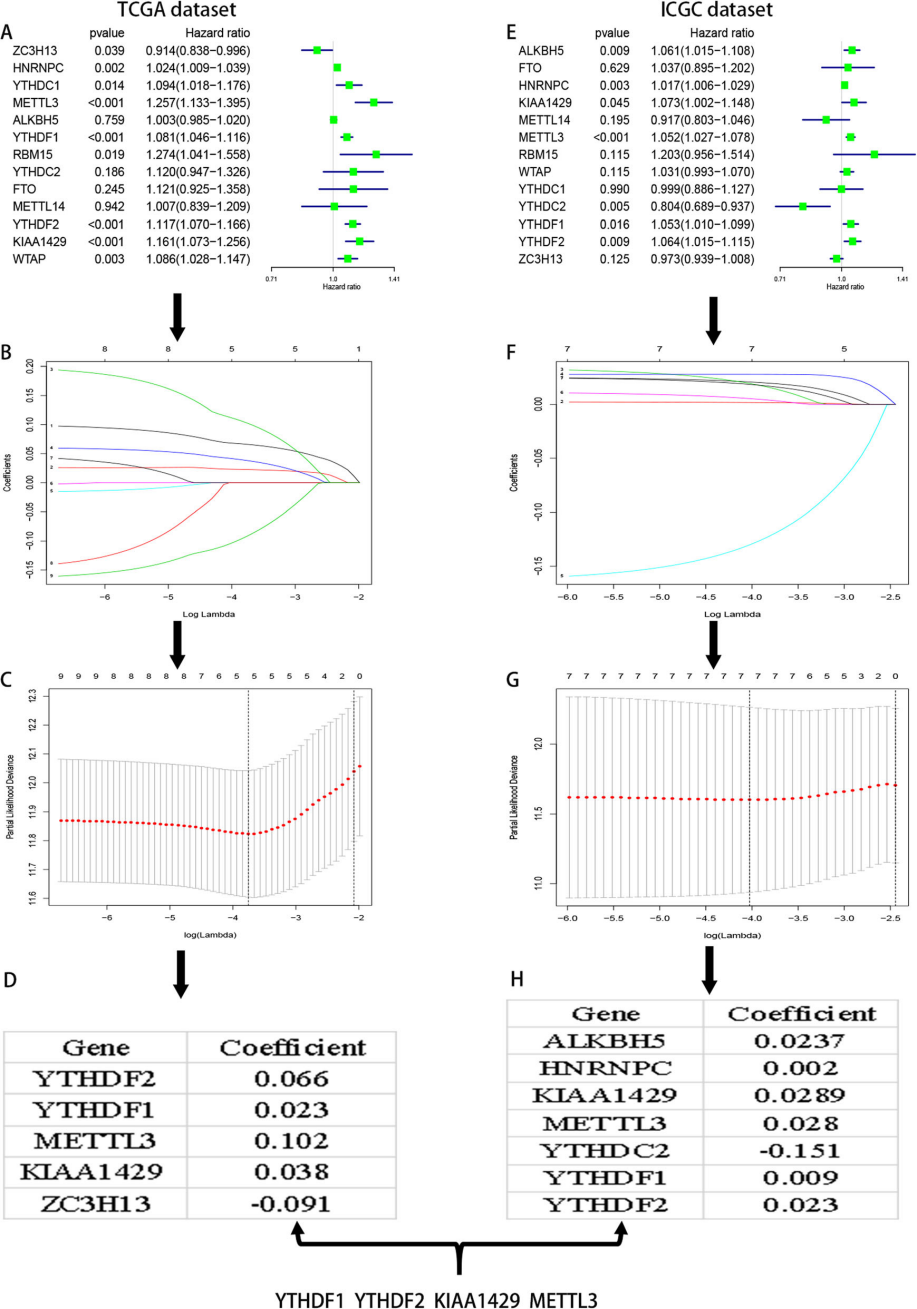
The process of selecting target genes to construct lasso risk regression model by applying the least absolute shrinkage and selection operator (LASSO) Cox regression algorithm. **a** Univariate Cox regression analysis of the 13 genes correlated with OS in TCGA dataset; the hazard ratios (HR), 95% confidence intervals .**c**-**d** The process of selecting target genes in TCGA dataset. **e**-**h** The process of selecting target genes in ICGC dataset

Next, we explored the prognostic value of m^6^A RNA methylation modulators in HCC. Univariate Cox regression analyses of TCGA data were performed to assess the associations between the expression levels of the 13 genes and OS (Fig. 3A). Nine genes were significantly associated with OS (*P* < 0.05). Among these nine genes, all but ZC3H13 were high-risk genes with hazard ratios (HRs) > 1, while ZC3H13 was a protective gene with HR < 1. We then used LASSO Cox regression to analyze the prognostic value of the nine genes in the TCGA dataset, and selected five genes based on the minimum criterion (Figs. 3B–D).

Additionally, univariate Cox regression analyses of ICGC data were performed to assess the associations between the expression levels of the 13 genes and OS (Fig. 3E). Seven genes were significantly associated with OS (*P* < 0.05). Among these seven genes, all but YTHDC2 were high-risk genes with HRs > 1, while YTHDC2 was a protective gene with HR < 1. Additionally, we used LASSO Cox regression to analyze the prognostic value of the seven genes in the ICGC dataset, and selected seven genes based on the minimum criterion (Figs. 3F–H).

Thereafter, we selected the common genes (YTHDF1, YTHDF2, METTL3, and KIAA1429) in the two database analyses to construct a four-gene risk signature based on the minimum criterion. We used the coefficients obtained using LASSO Cox regression to calculate prognostic risk scores for cases in both the training TCGA dataset (Figure S4A) and the validation ICGC dataset (Figure S4B).

### Risk scores showed strong associations with survival and clinicopathological factors in HCC

**Fig. 4:**
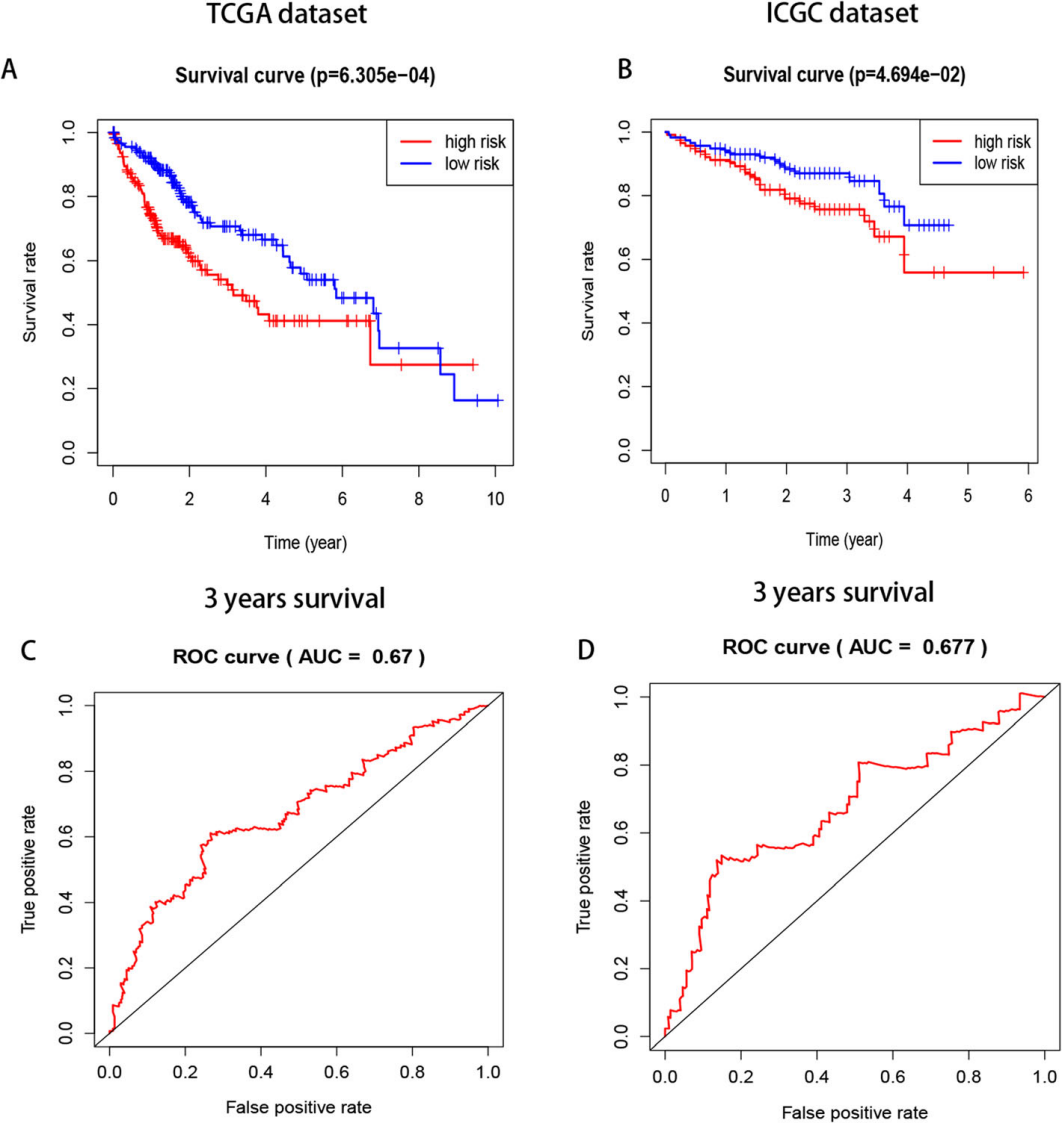
The differences in OS between the low-and high- risk groups based on the median risk score and ROC curve predict survival in TCGA and ICGC datasets. **a**-**b** significant differences in OS between the two categories (**c**-**d**) Kaplan–Meier overall survival (OS) curves for patients predicting 3 years survival in the TCGA (**c**) and ICGC (**d**) datasets assigned to high- and low-risk groups based on the risk score

Patients with HCC in the TCGA datasets were divided into low- and high-risk groups based on the median risk score, and a significant difference in OS was found between the two groups (Figs. 4A–B). Additionally, ROC curve analysis showed that the risk score could predict the 3-year survival rate of HCC patients (Figs. 4C–D).

**Fig. 5:**
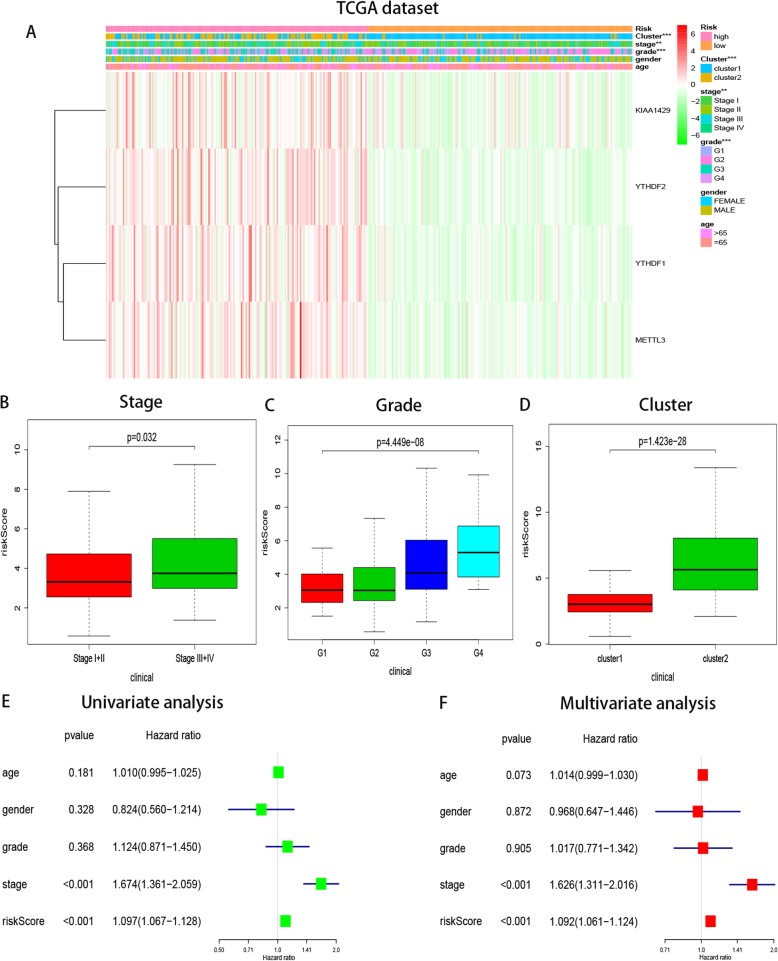
Relationship between the risk score, clinicopathological factors and cluster1/2 subgroups in TCGA dataset. **a** The heatmap shows the distribution of clinicopathological factors and four genes expression compared between the low- and high- risk groups. ** *P* < 0.01, *** *P* < 0.001. (**b**–**d**) Distribution of risk scores in the TCGA dataset stratified by WHO grade (**b**) pathological grade (**c**) and cluster1/2 subgroups (**d**). **e**-**f** Univariate and multivariate analyses of the association between clinicopathological factors (including the risk score) and overall survival of patients in the TCGA datasets, the hazard ratios (HR), 95% confidence intervals

The heatmap of the expression levels of the four selected m^6^A RNA methylation modulators in high- and low-risk patients in the TCGA dataset shows that the expression levels of the four genes were associated with increased risk scores (Figure S5A). Notably, survival rate and time were significantly reduced in the high-risk group compared to the low-risk group (Figure S5B). We constructed nomograms based on the risk score and clinicopathological factors to predict 1-, 3-, and 5-year survival rates (Figure S5C). Finally, we verified these trends using the ICGC data (Figures S6A–C).Next, using TCGA data, we conducted chi-square tests to determine whether we could better predict HCC clinical outcomes based on m6A RNA methylation modulators, and we found significant differences between low- and high-risk groups in WHO stage (*P* < 0.01), pathological grade (*P* < 0.001), and cluster 1/2 subgroups (*P* < 0.001, Fig. 5A). Chi-square tests showed that four genes were highly expressed in the high-risk group and decreased in the low-risk group. We explored the relationship between the risk score and each clinicopathological factor with Wilcoxon rank-sum tests. In the TCGA dataset, the higher the risk score, the higher the WHO stage and the worse the pathological grade (Figs. 5B–D); the risk score was higher in cluster 2 than in cluster 1.

Subsequently, univariate and multivariate Cox regression analyses of survival were performed using the risk score and relevant clinicopathological factors (age, sex, pathological grade, and WHO stage) in the TCGA dataset to determine whether the risk score (based on the four-gene risk signature) can be used as an independent prognostic factor. Both univariate and multivariate analyses showed that the risk score could be used as a prognostic indicator (both *P* < 0.001, Figs. 5E–F). WHO stage was also an independent prognostic indicator and the *P* value for age was near 0.05 (0.073), but sex and pathological grade were not independent prognostic indicators.

**Fig. 6:**
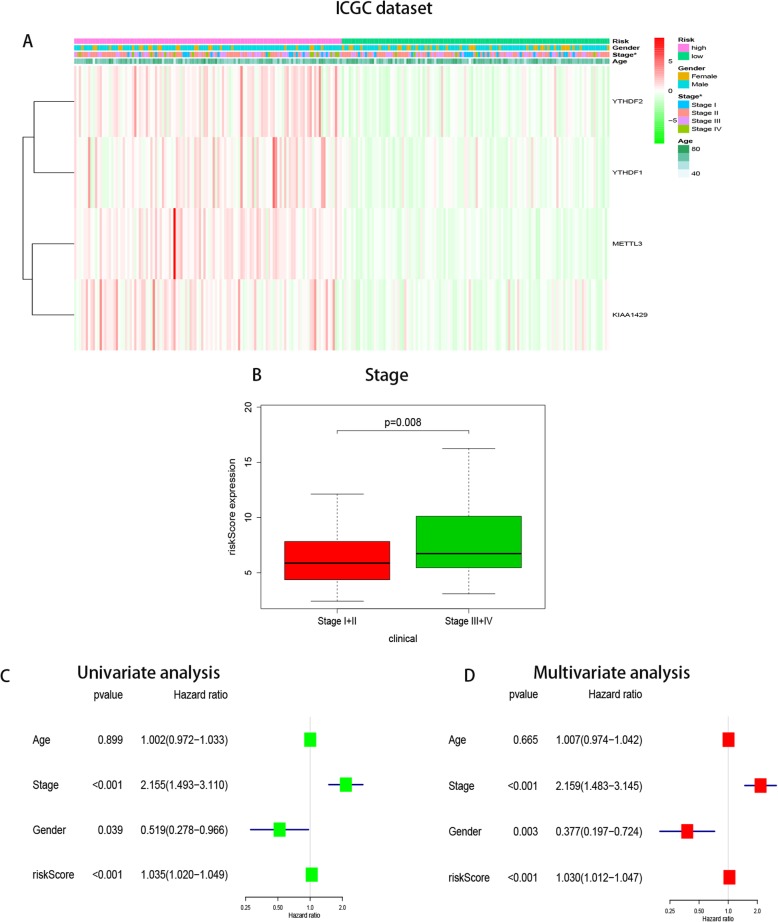
Relationship between the risk score and clinicopathological factors in ICGC dataset. **a** Distribution of risk scores in the TCGA dataset stratified by WHO grade. **b**-**c** Univariate and multivariate Cox regression analyses of the association between clinicopathological factors (including the risk score) and overall survival of patients in the ICGC dataset, the hazard ratios (HR), 95% confidence intervals

Additionally, we verified the results in the ICGC database. Owing to the lack of data on pathological grade, the relationships between the risk score and clinicopathological factors could not be fully investigated. However, the relationship between risk score and WHO stage was consistent with the TCGA analysis (*P* < 0.01, Fig. 6A). We also conducted univariate and multivariate regression analyses and, as in the TCGA analysis, the risk score and WHO stage were significantly associated with OS. Unlike in the TCGA analysis, there was a significant difference in sex in the ICGC analysis, with male sex leading to increased OS(*P* < 0.05 for both the univariate and multivariate analyses, Figs. 6B–C).

Furthermore, we constructed nomograms (involving risk score and clinicopathological factors) to predict 1-, 3-, and 5-year survival using TCGA (Figure S7A) and ICGC (Figure S7B) data. Regarding the TCGA nomograms, according to the above uni- and multivariate regression analyses using TCGA data, pathological grade was not an independent prognostic factor, so it was not included in the nomograms, while the *P* value for age in the multivariate regression analysis was 0.073 (close to 0.05), so it was included (Figure S7A). In the ICGC nomograms, age was included to be consistent with the TCGA nomograms. Sex was also included because it was a prognostic factor in the above uni- and multivariate regression analyses using ICGC data (Figure S7B).

## Discussion

HCC has been reported to be the second most common cause of cancer-related death worldwide [1]. HBV and HCV infections have resulted in a high rate of HCC in China. Because of the current lack of effective interventions, high metastasis rate, and high mortality rate, it is crucial to develop a deeper understanding of the molecular mechanism of HCC development. A growing body of evidence suggests that liver cancer is a multistep process associated with complex interactions between genetics, epigenetics, and transcriptional changes [32].

About 100 post-transcriptional chemical modifications can occur in biological RNAs [33] and m^6^A, which was discovered in the 1970s, is one of the most abundant endochemical modifications in eukaryotic mRNAs [8]. The biological significance of m^6^A RNA methylation has been increasingly recognized; it has important and diverse biological functions in mammals, including sex determination, tissue development, DNA damage response, circadian rhythm, and tumorigenesis [34]. In this study, we demonstrated that the expression of m^6^A RNA methylation modulators, which relates to the field of epigenetics, is also closely related to HCC prognosis. Based on the expression of m^6^A RNA methylation modulators, two subgroups of HCC tissues were identified by consensus clustering. These clusters had different prognoses and clinicopathological characteristics. In addition, we used four selected m^6^A RNA methylation modulators to derive a prognostic risk score, which was used to classify the HCC patients into high- and low-risk groups based on the median risk score.

Several studies have pointed out that the occurrence of liver cancer is related to the abnormal expression of m^6^A RNA methylation modulators [20, 25, 27–29, 35–37]. Zhao et al. [36] reported that increased YTHDF1 is related to poor prognosis of liver cancer patients, and YTHDF1 plays an important role in regulating liver cancer cell metabolism and cell cycle progression. Cheng and colleagues [27] reported that KIAA1429 facilitated HCC migration and invasion by inhibiting ID2 via increasing m^6^A modification of ID2 mRNA. Furthermore, Tanabe et al. [37] reported that YTHDC2 plays an important role in the growth of liver cancer cells.

The functions of METTL14, METTL3, and YTHDF2 in HCC are controversial. Ma et al. [20] demonstrated that METTL14 positively regulates the primary miRNA 126 (miR126) in an m^6^A-dependent manner by interacting with microprocessor complex subunit DiGeorge syndrome critical region 8 (DGCR8), and Ma et al. concluded that METTL14 can inhibit liver cancer metastasis. Ma et al. [20] also reported that METTL14 and m^6^A levels were decreased in HCC tissues compared with normal tissues or tissues adjacent to HCC tissues, while METTL3 and WTAP levels were basically unchanged, In contrast, Chen et al. [29] reported that METTL14 levels were slightly higher in liver cancer tissues than in normal tissues, and METTL3 levels were considerably higher. Based on this, Chen and colleagues concluded that both METTL14 and METTL3 play carcinogenic roles in HCC, and they are necessary for HCC growth and metastasis. Zhong et al. [25] reported that YTHDF2 may play an anti-tumor role in HCC because its overexpression inhibited cell proliferation and growth and promoted the apoptosis of HCC cells. In contrast, Yang et al. [28] and Chen et al. [29] found that YTHDF2 played a pro-cancer role in HCC. These studies indicate that abnormal expression of m^6^A RNA methylation modulators is closely related to HCC occurrence and development.

In this study, we comprehensively analyzed the expression of the 13 most common m^6^A RNA methylation modulators in HCC tissues. There was no difference in the expression of METTL14 between HCC and normal tissues from either database, which is consistent with the results reported by Zhou et al. [35]. Additionally, we analyzed the CNV data of these 13 genes using the TCGA database. The HCC tissues were significantly different from the normal tissues in terms of CNV. The expression of all 13 genes except HNRNPC was related to CNV. Only three genes (YTHDF1, YTHDC2, and KIAA1429) mainly exhibited increased copy numbers in the HCC tissues compared to the normal tissues, while the other 10 genes mainly exhibited reduced copy numbers. Furthermore, we analyzed data on SNP mutations in the 13 genes in HCC tissues and found very low mutation rates. These data indicate that abnormal expression of m^6^A RNA methylation modulators in HCC tissues is not entirely caused by genetic changes (CNV or SNP mutations) [38].

Whether the expression of m^6^A RNA methylation modulators can be used to predict cancer prognosis is an important research subject [7]. In this study, we used a risk signature constructed with four m^6^A RNA methylation modulators (YTHDF1, YTHDF2, KIAA1429, and METTL3) to predict OS among HCC patients. In the TCGA database, patients with high risk scores ​​were more likely to have a higher WHO stage and higher pathological grade and be in HCC subtype cluster 2. Also, in the ICGC database, high risk scores were associated with higher WHO stage. It should be noted that the risk score was independently associated with OS among HCC patients in both the TCGA and ICGC analyses. However, unlike in the TCGA analysis, sex was also an independent prognostic factor for HCC in the ICGC analysis, which may have been due to ethnic differences between the two datasets.

The METTL3 RNA methyltransferase is a “writer” protein responsible for m^6^A modification and is involved in mRNA biogenesis, decay, and translation. METTL3 may play a carcinogenic role in lung cancer [39], bladder cancer [23, 40], gastric cancer [41], osteosarcoma [42], cutaneous squamous cell carcinoma [43], and acute myeloid leukemia (AML) [44]. Li et al. [45] reported that METTL3 promoted colorectal cancer progression through an m^6^A-IGF2BP2-dependent mechanism, while Deng et al. [46] reported that METTL3 inhibited the proliferation and migration of colorectal cancer cells through the p38/ERK pathway. Additionally, Cui and colleagues [47] reported that METTL3 downregulation significantly promoted the growth, self-renewal, and tumorigenesis of human glioblastoma stem cells, while METTL3 overexpression inhibited the growth and self-renewal of these cells. However, Visvanathan et al. [48] reported that METTL3 transcription was increased in glioblastoma, while METTL3 silencing inhibited tumor growth and prolonged the survival of mice. These results suggest that METTL3 may play varied roles in different types of cancer, and the study of METTL3 in colorectal cancer and glioblastoma remains controversial.

As a component of the m^6^A “writer” complex, KIAA1429 is reported to be an RNA-binding protein involved in m^6^A modification and RNA splicing and processing. At present, its role as an m^6^A “writer” in tumorigenesis and its mechanism have not been fully reported. However, Cheng et al. [27] reported that KIAA1429 inhibits ID2 by increasing the m^6^A modification of ID2 mRNA, thus promoting HCC migration and invasion. Additionally, Qian and colleagues [24] reported that KIAA1429 can regulate CDK1 in breast cancer in an m^6^A-independent manner, and act as a carcinogenic factor. These studies suggest that KIAA1429 promotes tumorigenesis and development.

YTH N^6^-methyladenosine RNA binding protein 1 (YTHDF1) is a member of the YTH domain family, which includes YTHDF1, 2, and 3 and YTHDC1 and 2. As a “reader” of m^6^A-modified mRNA, cytoplasmic YTHDF1 interacts with binding sites on m^6^A-modified mRNA to promote the initiation of translation [49]. However, the link between YTHDF1 and cancer is largely unknown. Han et al. [50] reported that m^6^A RNA modification, involving YTHDF1, modulates the anti-tumor immune response. Zhao et al. [36] and Zhou et al. [35] reported that YTHDF1 is highly expressed in liver cancer and is significantly associated with poor prognosis. Furthermore, Nishizawa et al. [51] and Bai et al. [22] reported that YTHDF1 is highly expressed in colorectal cancer and plays an important role in carcinogenesis.

The main role of YTHDF2 is to regulate the degradation of m^6^A-modified mRNAs [24]. However, the relationship between YTHDF2 and cancer is largely unknown. Yang et al. [28] reported that miR-145 regulates m^6^A by targeting the 3′-untranslated region of YTHDF2 in HCC cells, and YTHDF2 expression is closely related to the malignant degree of HCC. Thereafter, Chen and colleagues [52] reported that YTHDF2 was highly expressed in pancreatic cancer, which promoted the proliferation and inhibited the migration and invasion of pancreatic cancer cells. Furthermore, many studies have focused on the relationship between YTHDF2 and AML, indicating that YTHDF2 is increased in the broad spectrum of human AML tissues. Targeting YTHDF2 to inhibit its expression can enlarge hematopoietic stem cells, enhance bone marrow reconstruction, and selectively impair AML [53–55], suggesting that it may be useful in the treatment of hematological malignant tumors.

## Conclusions

Our results systematically demonstrate the expression of 13 m^6^A RNA methylation modulators in HCC, reveal the CNV and SNP mutations of these genes in HCC, and clarify the prognostic value of the m^6^A RNA methylation modulators. Our study provides important information for further exploration of the role of m^6^A RNA methylation in HCC.

## Supplementary information

**Additional file 1: Figure S1.** CNV copy number variation and SNP mutations of m6A RNA methylation modulators in HCC. (A) CNV copy number variation of the 13 genes in HCC tissues. Dioploid means normal; Shallow deletion means deletion of one copy number; Copy number gain means get a copy number; Amplification means get two or more copies. (B) SNP mutations of the 13 genes in HCC tissues. The vertical coordinate represents each gene, and the horizontal coordinate represents the number and type of SNP mutations of each gene in 364 tumor samples.

**Additional file 2: Figure S2.** The relationship between mRNA expression of the 13 genes and copy number. (A-M) Kruskal-Wallis test was used for the differences among the groups, Single deletion means deletion of one copy number; Single gain means get a copy number; Amplification means get two or more copies.The numbers in the figures represent the sample size for each category of CNV.

**Additional file 3: FigureS3.** Consensus clustering of m6A RNA methylation modulators identified two clusters of hepatocellular carcinoma and gene differential expression between two clusters. (A)Consensus clustering cumulative distribution function (CDF) for k = 2 to 9. (B) Relative change in area under CDF curve for k = 2 to 9. (C-D) Consensus clustering matrix for k = 2(C) and k = 3(D). (E) The tracking plot for k = 2 to k = 9. (F) Heatmap of expression of m6A RNA methylation modulators of the two clusters, gene differential expression was tested by Wilcox test, * *P* < 0.05 and *** *P* < 0.001.

**Additional file 4: Figure S4.** Risk signature with four common m6A RNA methylation modulators. (A-B) The process of building the signature containing four m6A RNA methylation modulators in two datasets. The hazard ratios (HR), 95% confidence intervals (CI) calculated by univariate Cox regression and the coefficients calculated by multivariate Cox regression using LASSO are shown.

**Additional file 5: Figure S5.** Lasso risk regression model risk diagram in TCGA dataset. (A) Heatmap of the expression levels of the four selected m6A RNA methylation modulators in high- and low-risk patients. (B) Survival status map, the green dots on behalf of survival state, the red dots on behalf of death state. (C)Nomograms based on the risk score and clinicopathological factors to predict 1-, 3-, and 5-year survival rates.

**Additional file 6: Figure S6.** Lasso risk regression model risk diagram in ICGC dataset. (A) Heatmap of the expression levels of the four selected m6A RNA methylation modulators in high- and low-risk patients. (B) Survival status map, the green dots on behalf of survival state, the red dots on behalf of death state. (C)Nomograms based on the risk score and clinicopathological factors to predict 1-, 3-, and 5-year survival rates.

**Additional file 7: Figure S7.** Nomograms combining risk values with clinicopathological factors to predict survival. (A) A comprehensive nomogram composed of age and stage combined with risk values, was used to predict 1-year, 3-year and 5-year survival rates in the TCGA database. (B)A comprehensive nomogram composed of age, stage and gender combined with risk values was used to predict the 1-year, 3-year and 5-year survival rate In the ICGC database.

**Additional file 8: Table S1.** Summary of expression and genetic changes of m6A RNA methylation modulators in HCC.

**Additional file 9: Table S2.** Clinicopathological features of patients included in this study.

## Data Availability

All analyzed data are included in this published article and its supplementary information file. The original data are available upon request to the corresponding author.

## Abbreviations

AML: Acute myeloid leukemia
CNV: Copy number variation
HBV: Hepatitis B virus
HCC: Hepatocellular carcinoma
HCV: Hepatitis C virus
HR: Hazard ratio
HTSeq: High-throughput sequencing
ICGC: International Cancer Genome Consortium
LASSO: Least absolute shrinkage and selection operator
miRNA: microRNA
mRNA: Messenger RNA
OS: Overall survival
PCA: Principal component analysis
ROC: Receiver operating characteristic
SNP: Single-nucleotide polymorphism
TCGA: The Cancer Genome Atlas
WHO: World Health Organization
YTHDF: YTH N^6^-methyladenosine RNA binding protein
