# Supplementary figures and images for: Multiple m6A RNA methylation modulators promote the malignant progression of hepatocellular carcinoma and affect its clinical prognosis
Source: BMC Cancer. 2020 Feb 28;20:165. doi: 10.1186/s12885-020-6638-5 (PMC7047390; doi:10.1186/s12885-020-6638-5)

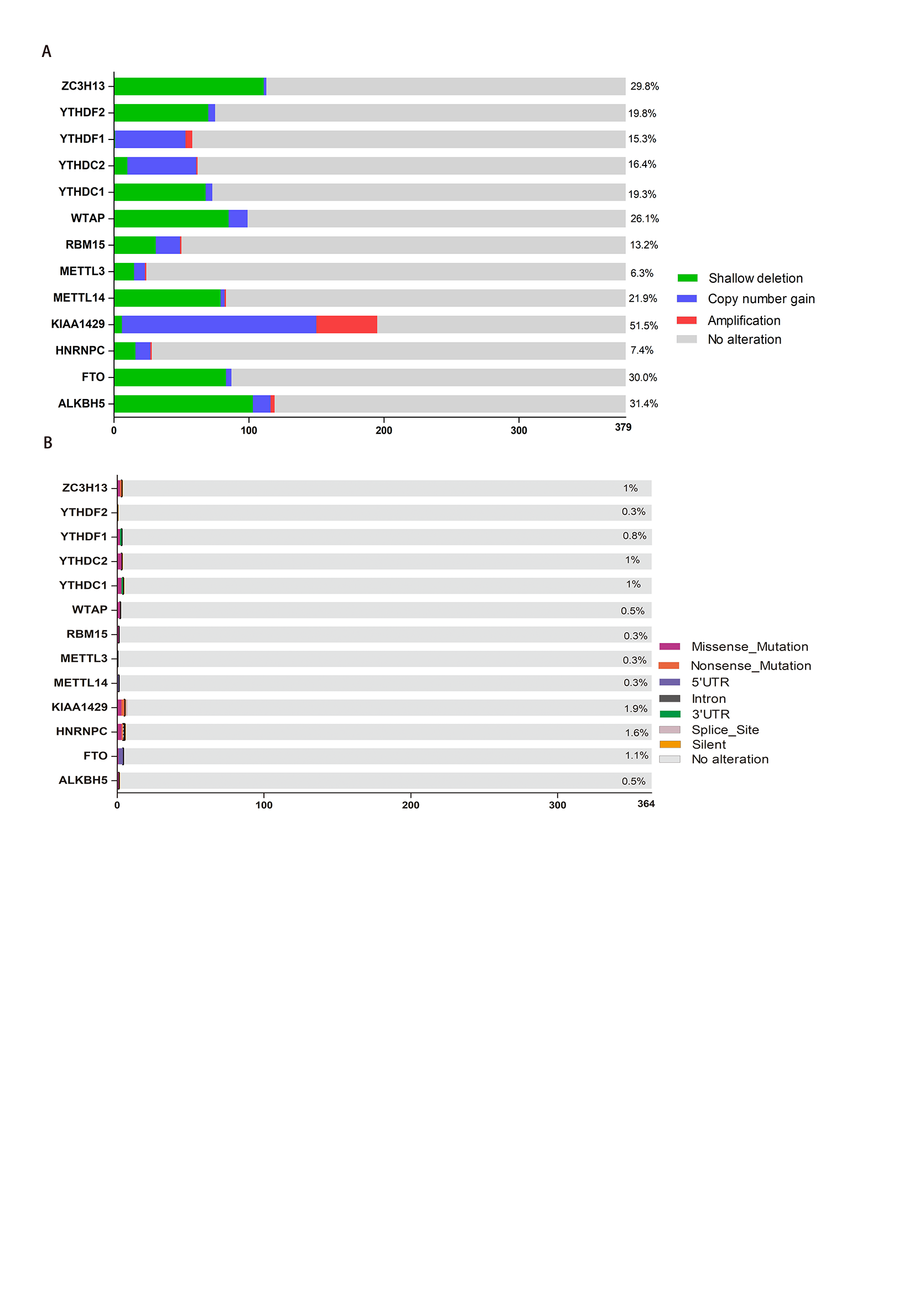

Supplement: Supplementary file 1 — Additional file 1: Figure S1. CNV copy number variation and SNP mutations of m6A RNA methylation modulators in HCC. (A) CNV copy number variation of the 13 genes in HCC tissues. Dioploid means normal; Shallow deletion means deletion of one copy number; Copy number gain means get a copy number; Amplification means get two or more copies. (B) SNP mutations of the 13 genes in HCC tissues. The vertical coordinate represents each gene, and the horizontal coordinate represents the number and type of SNP mutations of each gene in 364 tumor samples. [file 12885_2020_6638_MOESM1_ESM.png]

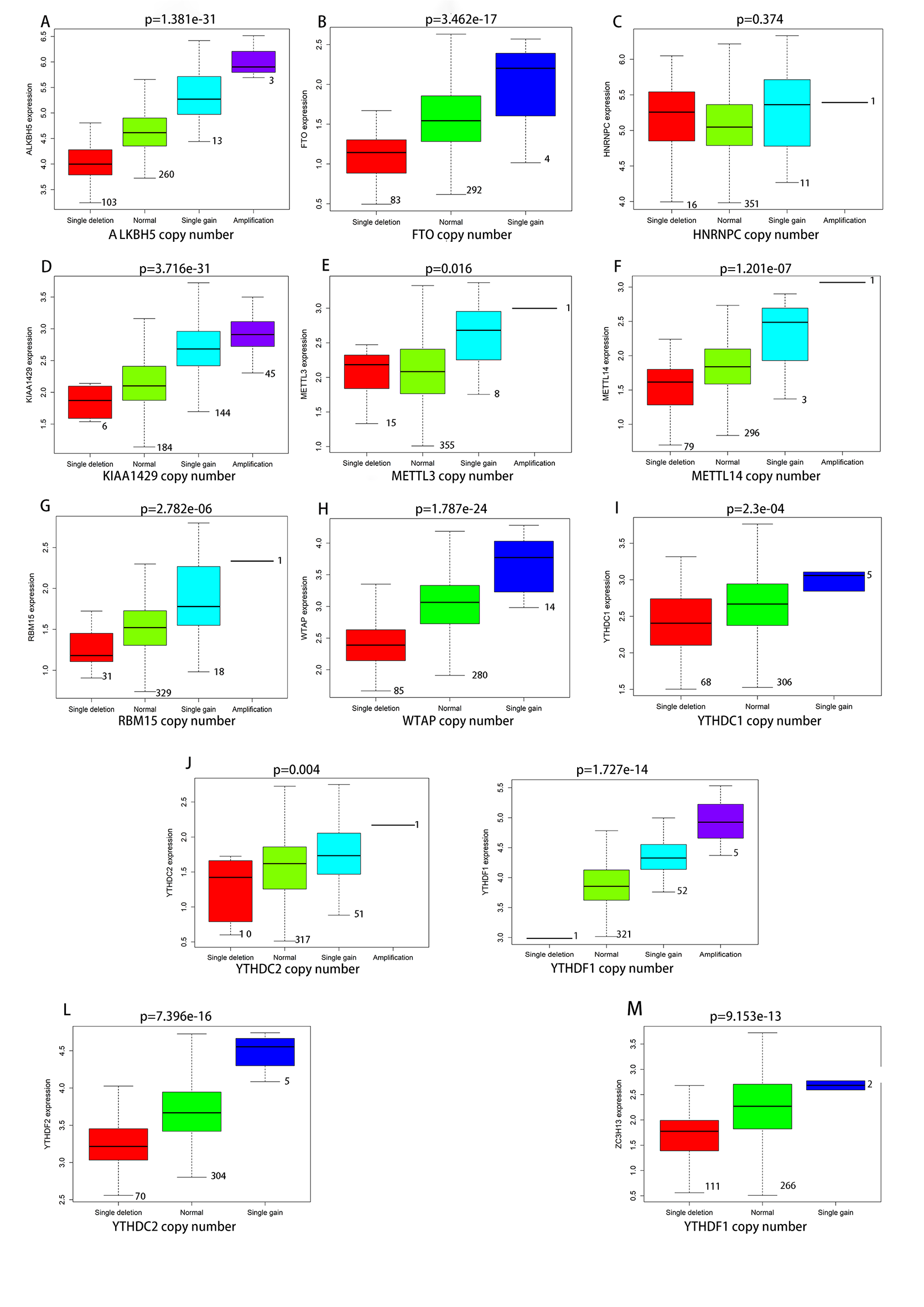

Supplement: Supplementary file 2 — Additional file 2: Figure S2. The relationship between mRNA expression of the 13 genes and copy number. (A-M) Kruskal-Wallis test was used for the differences among the groups, Single deletion means deletion of one copy number; Single gain means get a copy number; Amplification means get two or more copies.The numbers in the figures represent the sample size for each category of CNV. [file 12885_2020_6638_MOESM2_ESM.tif]

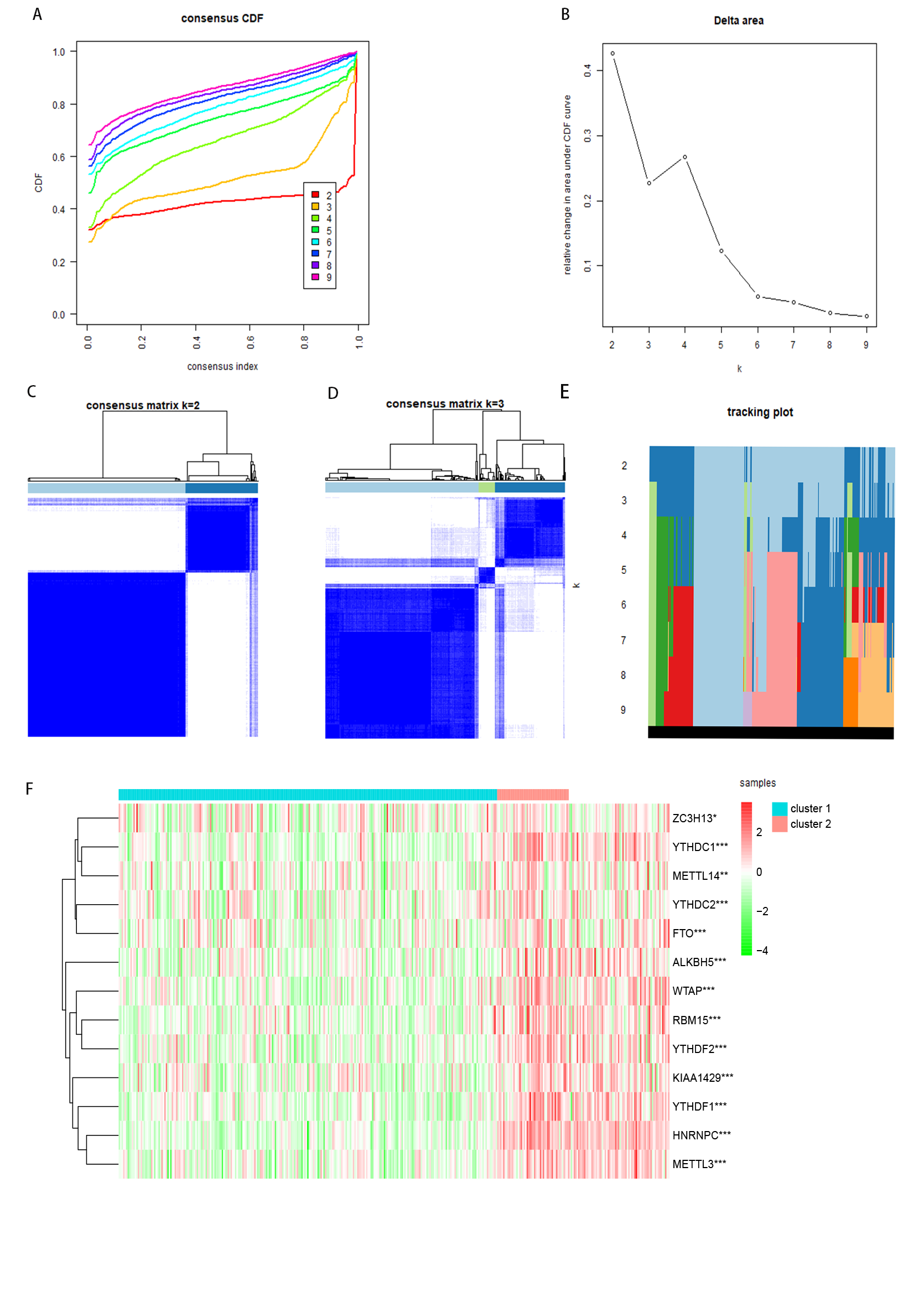

Supplement: Supplementary file 3 — Additional file 3: FigureS3. Consensus clustering of m6A RNA methylation modulators identified two clusters of hepatocellular carcinoma and gene differential expression between two clusters. (A)Consensus clustering cumulative distribution function (CDF) for k = 2 to 9. (B) Relative change in area under CDF curve for k = 2 to 9. (C-D) Consensus clustering matrix for k = 2(C) and k = 3(D). (E) The tracking plot for k = 2 to k = 9. (F) Heatmap of expression of m6A RNA methylation modulators of the two clusters, gene differential expression was tested by Wilcox test, * P < 0.05 and *** P < 0.001. [file 12885_2020_6638_MOESM3_ESM.tif]

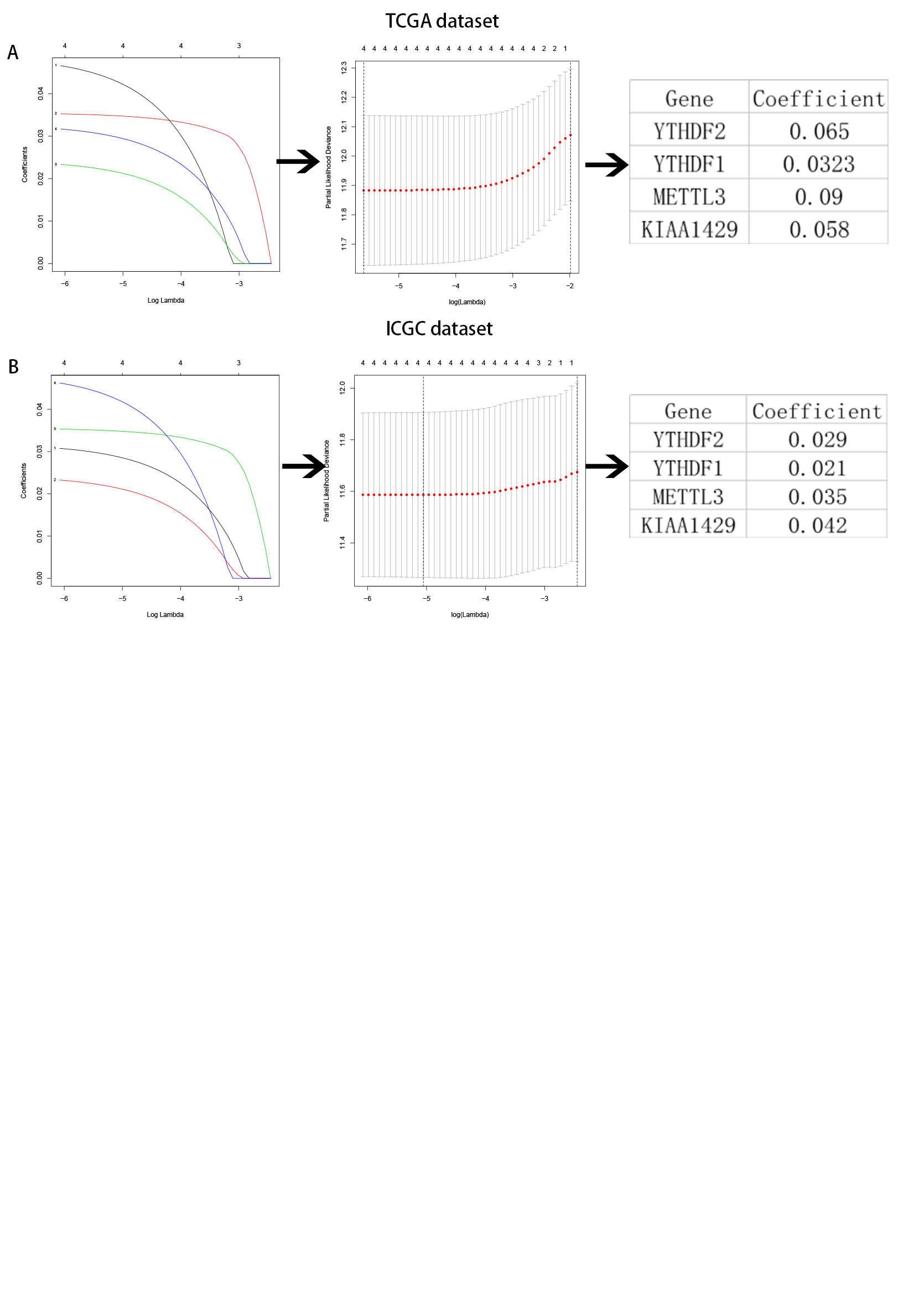

Supplement: Supplementary file 4 — Additional file 4: Figure S4. Risk signature with four common m6A RNA methylation modulators. (A-B) The process of building the signature containing four m6A RNA methylation modulators in two datasets. The hazard ratios (HR), 95% confidence intervals (CI) calculated by univariate Cox regression and the coefficients calculated by multivariate Cox regression using LASSO are shown. [file 12885_2020_6638_MOESM4_ESM.tif]

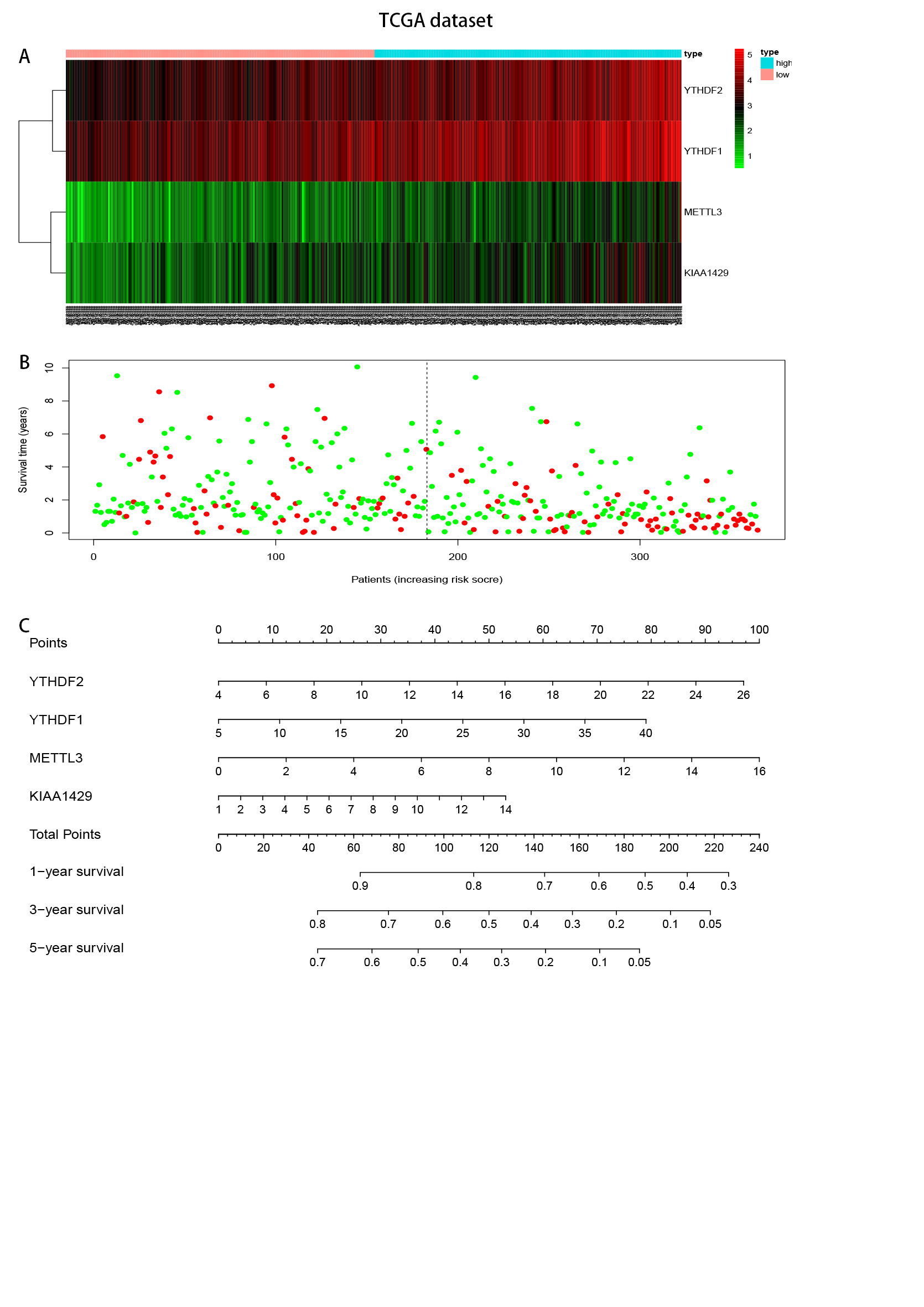

Supplement: Supplementary file 5 — Additional file 5: Figure S5. Lasso risk regression model risk diagram in TCGA dataset. (A) Heatmap of the expression levels of the four selected m6A RNA methylation modulators in high- and low-risk patients. (B) Survival status map, the green dots on behalf of survival state, the red dots on behalf of death state. (C)Nomograms based on the risk score and clinicopathological factors to predict 1-, 3-, and 5-year survival rates. [file 12885_2020_6638_MOESM5_ESM.tif]

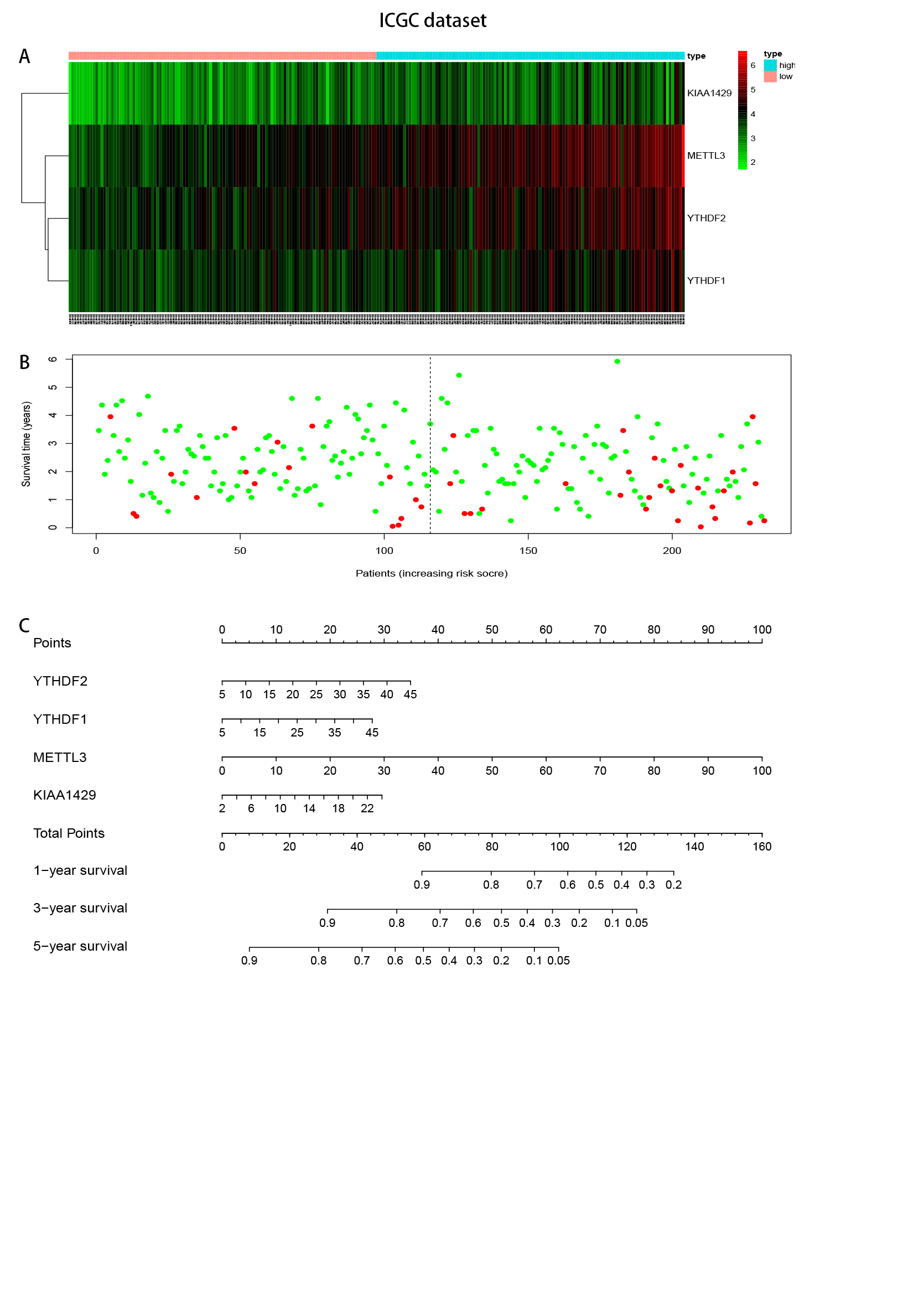

Supplement: Supplementary file 6 — Additional file 6: Figure S6. Lasso risk regression model risk diagram in ICGC dataset. (A) Heatmap of the expression levels of the four selected m6A RNA methylation modulators in high- and low-risk patients. (B) Survival status map, the green dots on behalf of survival state, the red dots on behalf of death state. (C)Nomograms based on the risk score and clinicopathological factors to predict 1-, 3-, and 5-year survival rates. [file 12885_2020_6638_MOESM6_ESM.tif]

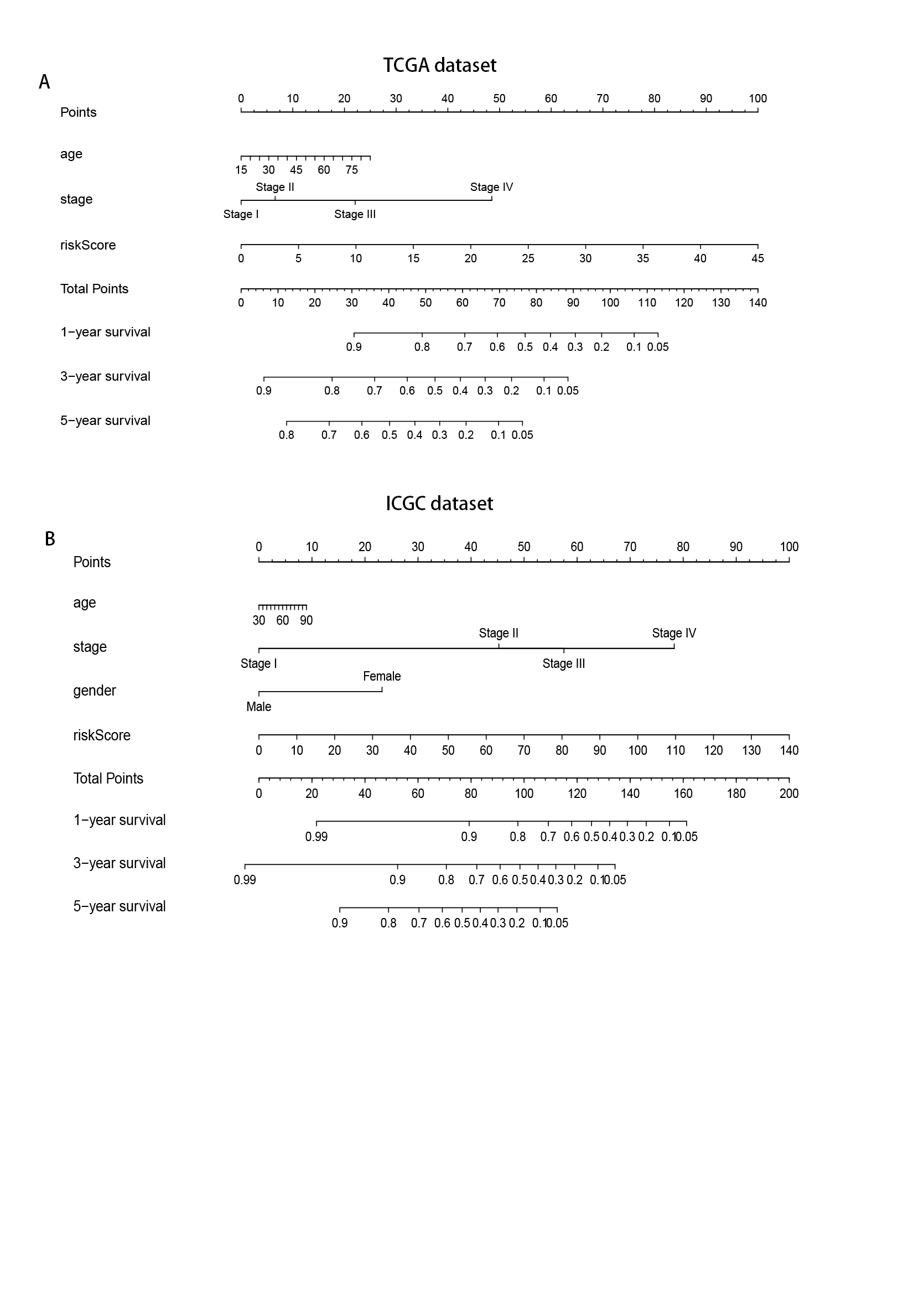

Supplement: Supplementary file 7 — Additional file 7: Figure S7. Nomograms combining risk values with clinicopathological factors to predict survival. (A) A comprehensive nomogram composed of age and stage combined with risk values, was used to predict 1-year, 3-year and 5-year survival rates in the TCGA database. (B)A comprehensive nomogram composed of age, stage and gender combined with risk values was used to predict the 1-year, 3-year and 5-year survival rate In the ICGC database. [file 12885_2020_6638_MOESM7_ESM.tif]
