# Supplementary material for: Multiple m6A RNA methylation modulators promote the malignant progression of hepatocellular carcinoma and affect its clinical prognosis
Source: BMC Cancer. 2020 Feb 28;20:165. doi: 10.1186/s12885-020-6638-5 (PMC7047390; doi:10.1186/s12885-020-6638-5)
Supplement: Supplementary file 8 — Additional file 8: Table S1. Summary of expression and genetic changes of m6A RNA methylation modulators in HCC. [file 12885_2020_6638_MOESM8_ESM.doc]

**Table S1. Summary of expression and genetic changes of m6A RNA methylation modulators in HCC**

| **Gene** | **Developed names** | **Functions** | **Locations [Homo sapiens]** | **Expression in TCGA(tumor vs normal)** | **Expression in ICGC(tumor vs normal)** | **Major CNV(tumor vs normal)** | **The relationship between**  **expression and CNV** | **SNP**  **(tumor vs normal)** |
| --- | --- | --- | --- | --- | --- | --- | --- | --- |
| METTL3 | IME4,M6A,MT-A70, Spo8, hMETTL3 | Writers | Chromosome 14, NC_000014.9 | Up | Up | Decreased copy numbers | Related | - |
| METTL14 | hMETTL14 | Writers | Chromosome 4, NC_000004.12 | - | - | Decreased copy numbers | Related | - |
| WTAP | Mum2 | Writers | Chromosome 6, NC_000006.12 | Up | Up | Decreased copy numbers | Related | - |
| KIAA1429 | [VIRMA](https://www.ncbi.nlm.nih.gov/gene/25962)、MSTP054, fSAP121 | Writers | Chromosome 8, NC_000008.11 | Up | Up | Increased copy numbers | Related | - |
| ZC3H13 | KIAA0853, Xio | Writers | Chromosome 13, NC_000013.11 | - | Up | Decreased copy numbers | Related | - |
| RBM15 | OTT, OTT1, SPEN | Writers | Chromosome 1, NC_000001.11 | Up | Up | Decreased copy numbers | Related | - |
| FTO | ALKBH9, BMIQ14, GDFD | Erasers | Chromosome 16, NC_000016.10 | Up | Up | Decreased copy numbers | Related | - |
| ALKBH5 | ABH5, OFOXD, OFOXD1 | Erasers | Chromosome 17, NC_000017.11 | Up | Up | Decreased copy numbers | Related | - |
| YTHDF1 | C20orf21 | Readers | Chromosome 20, NC_000020.11 | Up | Up | Increased copy numbers | Related | - |
| YTHDF2 | CAHL, HGRG8, NY-REN-2 | Readers | Chromosome 1, NC_000001.11 | Up | Up | Decreased copy numbers | Related | - |
| YTHDC1 | YT521, YT521-B | Readers | Chromosome 4, NC_000004.12 | Up | Up | Decreased copy numbers | Related | - |
| YTHDC2 | CAHL, hYTHDC2 | Readers | Chromosome 5, NC_000005.10 - | Up | Up | Increased copy numbers | Related | - |
| HNRNPC | C1, C2, HNRNP, HNRPC, SNRPC | Readers | Chromosome 14, NC_000014.9 | UP | Up | Decreased copy numbers | - | - |
