# Supplementary material for: Multiple m6A RNA methylation modulators promote the malignant progression of hepatocellular carcinoma and affect its clinical prognosis
Source: BMC Cancer. 2020 Feb 28;20:165. doi: 10.1186/s12885-020-6638-5 (PMC7047390; doi:10.1186/s12885-020-6638-5)
Supplement: Supplementary file 9 — Additional file 9: Table S2. Clinicopathological features of patients included in this study. [file 12885_2020_6638_MOESM9_ESM.doc]

**Table S2. Clinicopathological features of patients included in this study.**

|  | **TCGA dataset** | | **ICGC dataset** | |
| --- | --- | --- | --- | --- |
|  | Number | Percentage | Number | Percentage |
| **Total** | 350 | 100.00% | 260 | 100.00% |
| **Age** | 16-85 |  | 31-89 |  |
| ≤65 | 227 | 64.9% | 98 | 37.7% |
| >65 | 123 | 35.1% | 162 | 62.3% |
| **Gender** Female | 111 | 31.7% | 68 | 26.2% |
| Male | 239 | 68.3% | 192 | 73.8% |
| **WHO Stage** I | 174 | 49.7% | 40 | 15.4% |
| II | 85 | 24.3% | 117 | 45.0% |
| III | 86 | 24.6% | 80 | 30.8% |
| IV | 5 | 1.4% | 23 | 8.8% |
| **[Pathological](../../../../C:/Program%20Files%20(x86)/Youdao/Dict/8.5.3.0/resultui/html/index.html#/javascript:;)** **Grade** G1 | 46 | 13.1% | - | - |
| G2 | 171 | 48.9% | - | - |
| G3 | 120 | 34.3% | - | - |
| G4 | 13 | 3.7% | - | - |
